# Monitoring the Residual Limb-Socket Interface: A Perspective on Clinical Needs and Challenges

**DOI:** 10.1007/s44174-025-00594-w

**Published:** 2025-12-28

**Authors:** Ming Liu, Brendan Evert, Sooyoung Kim, Michael D. Dickey, Alper Bozkurt, William Filer

**Affiliations:** 1 Lampe Joint Department of Biomedical Engineering, University of North Carolina – Chapel Hill, Chapel Hill, USA; 2 Lampe Joint Department of Biomedical Engineering, North Carolina State University, Raleigh, USA; 3 Department of Electrical and Computer Engineering, North Carolina State University, Raleigh, USA; 4 Department of Chemical and Biomolecular Engineering, North Carolina State University, Raleigh, USA; 5 Department of Physical Medicine and Rehabilitation, University of North Carolina, Chapel Hill, USA

**Keywords:** lower limb amputee, Prosthetic socket, Skin health, Wearable sensor

## Abstract

Skin-related complications are among the most common secondary conditions experienced by lower limb amputees. Besides connecting the prosthetic legs, the residual limb–socket interface (RLSI) plays a crucial role in maintaining skin health for prosthesis users. However, RLSI evaluation typically relies on subjective feedback from amputees, who may have difficulty accurately assessing the condition of the interface—particularly those with sensory impairments. RLSI monitoring systems have the potential to improve care by providing objective, real-time, and quantitative feedback to amputees, prosthetists, and clinicians. Despite advancements in sensing technologies, most RLSI monitoring research has focused on technical development, with limited attention to clinical integration. As a result, there is a lack of clinical evidence demonstrating improved outcomes from using these monitoring systems. This perspective aims to help researchers align their sensing technologies with current clinical practices so that RLSI monitoring data can support evidence-based medical decision-making. We begin by reviewing RLSI manufacturing and maintenance procedures, followed by an overview of common clinical strategies used to optimize RLSI performance. Next, we discuss the challenges of incorporating RLSI monitoring systems into existing clinical workflows. Finally, we identify key clinical needs and highlight several promising monitoring technologies that could address those needs.

## Introduction

Prosthetic legs are critical clinical tools for lower limb amputees, and the residual limb-socket interface (RLSI) is the most feasible interface for amputees to connect with their prosthetic legs. Made of a well-fitted prosthetic socket and other optional components, a reliable RLSI must with-stand repeatedly dynamical loads and provide reliable support and protection. Failures of this interface often lead to physical, mental, and financial damage to amputees. To enhance the performance of RLSI, various RLSI monitoring systems have been developed and some of them have been commercially available for more than 20 years.

Despite the advantage of technological development, the clinical application of these monitoring systems is still very limited, and their clinical impacts are trivial. In addition to the fact that most RLSI monitoring systems are only prototypes, two major barriers exist: (1) researchers and engineers focus on technology development and are unfamiliar with clinical practice, so some key issues, such as cost, usability, and reliability, are not fully considered; and (2) clinicians/patients have difficulty interpreting sensing system measurements and need additional guidance to link these measurements to their clinical practice.

Rather than surveying the latest RLSI technologies, this perspective focuses on outlining standard clinical procedures and clarifying real-world clinical needs. The goal is to help engineers overcome translational barriers by adapting their technologies to align with clinical workflows and initiating studies that establish evidence-based procedures for using RLSI monitoring data. We also identify several persistent clinical challenges and highlight emerging technologies that, based on the authors’ understanding of clinical practice, have the potential to address these challenges.

## Challenges for Lower Limb Amputee Rehabilitation Caused by the Residual Limb-Socket Interface (RLSI)

Lower limb amputation is a major source of disability in both the developing and developed world [1-5]. Approximately 150,000 major lower limb amputations are conducted in the US alone [6], and the average lifelong health care costs for an amputee in 2024 can be over $800,000 when accounting for inflation [7]. Prosthetic legs are key assistance devices used by lower limb amputees to regain upright locomotion. A higher level of prosthetic leg usage is often related to better rehabilitation outcomes, such as a higher employment rate [8] and better quality of life [9].

A critical issue for prosthesis users is the comfortable and reliable mounting of prostheses on their residual limb. Although the fusion of a prosthesis connection directly to bone (osseointegration) is now clinically available in multiple countries, its availability remains limited, and long-term clinical impacts are still under investigation [10]. Traditional prosthetic sockets remain the most widely available interfaces to connect prosthetic legs for lower limb amputees.

**Fig. 1:**
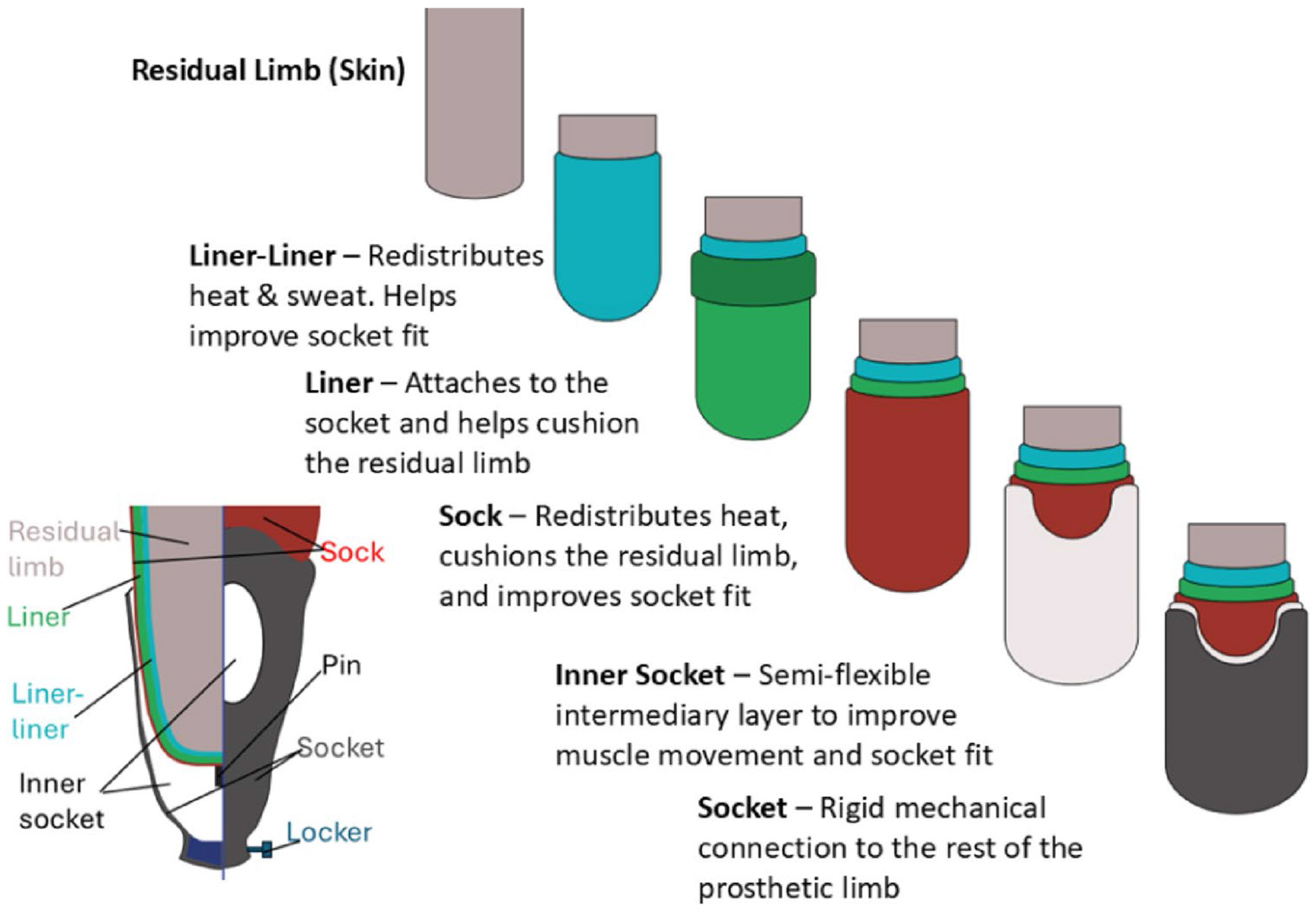
An example of the residual limb prosthetic socket interface (with pin-lock). In addition to the socket, including the inner socket, other items, which are used to enhance the reliability of the interface, such as liner, liner-liner, sock, and pin-locker, form the suspension system

Connecting prosthetic legs with residual limbs through prosthetic sockets involves multiple subsystems. Here, we provide a very brief introduction to some common methods based on a transtibial system as an example as shown in Fig. 1 with a pin-lock suspension design. *First*, a prosthetic socket is the core component used to connect the residual limb and prosthetic leg. It is usually made of materials with high strength/weight ratio, such as carbon fiber, and mimics the shape of the residual limb. An inner socket (an optional item), which is made of softer material than the rigid carbon fiber, can also be included as an internal layer of the socket. *Second*, suspension systems are introduced to stabilize the interface between the residual limb and provide cushioning within the prosthetic socket. Liners are introduced to generate more comfortable connections with the sockets. Prosthetic socks are included, so amputees can don/doff them to compensate for volume changes in the residual limb. *Finally*, additional components ensure reliable connection of prosthetic sockets, especially during leg swing. These include waist belts, pin-lock systems on the socket (as shown in Fig. 1), and sleeves, which help maintain an air seal between the residual limb and the socket. Other components, which are not on the prosthetic socket, can also contribute to the reliable connection between the residual limb and the socket, such as an air pump on the foot designed to enhance vacuum between the residual limb and the socket [11].

RLSI design can vary significantly in real clinical applications. Except for the socket, other components are all optional and clinicians need to choose the appropriate combination based on amputees’ status. A more detailed description of the prosthetic socket, related suspension systems, and components can be found in [12, 13]. It is also necessary to point out that the level of amputation affects the socket design principles and selection of components. Special concerns related to transtibial, transfemoral, hip disarticulation, and hemipelvectomy can be found in [14-17].

### Negative Clinical Impact of Poor RLSI

Pressure ulcers and related skin issues are the most direct consequences. Approximately 63% of lower limb amputees report skin issues [18], which is the number one secondary condition resulting from lower limb amputation [19]. Amputees experiencing skin ulcers need multiple clinic visits for evaluation, prosthesis modifications and injury treatments. They face additional risk of infection or revision surgery and are frequently requested to give up their prosthetic device while the ulcer heals. Treatment and recovery procedures also impose additional financial and psychological burdens on amputees suffering from these conditions.

Poor RLSI also triggers amputees to adopt additional compensation efforts to reduce loads on the residual limb in stance or hold RLSI in swing. These abnormal compensation efforts reduce walking speed, increase mental load during locomotion, and the generated abnormal gait pattern makes it more challenging to maintain balance and can cause injuries at other joints. As a result, socket fits and related skin issues are still the main reasons why lower limb amputees abandon their prosthetic legs [20].

Different levels of amputation also bring in unique clinical challenges. For example, transfemoral sockets often reduce the range of motion at hip joints [21] and hip disarticulation or hemipelvectomy amputees often suffers socket dislocation, which causes injuries and discomforts [22].

### Clinicians’ Challenges

The design, fitting and maintenance of the *RLSI presents substantial challenges*, *which usually reflect on prosthetic sockets*, *the key components of the RLSI*, for clinicians. A prosthetic socket must successfully transfer loads from the socket to the residual limb without creating pressure or shear forces that will cause pain or skin breakdown. It must also provide a means of suspending the leg against gravity and centrifugal force during swing. Designers must consider the following: (1) the shapes of residual limbs vary significantly among amputees; (2) soft tissues are unevenly distributed on the residual limb and deform under mechanical loads; (3) walking is highly dynamic and generates time-varying changes in pressure distributions; (4) a gradual or sudden change in residual limb shape is possible; *(5) unique consideration based on the level of amputations*, and (6) there are many user-specific challenges, such as the presence of scar tissue, sweating, and sensory changes [23]. *Although other parts of the RLSI are often standard components and cost less*, *clinicians also need to make evidenced clinical decisions*, *such as selecting different types of suspension systems*, *adjusting types of liners*, *and even deciding setting for air pumps.*

Clinicians have few tools available to quantify the quality of the RLSI, leading prosthetists to rely on an iterative trial-and-error approach to fit a socket. First, patients are given a socket to have a brief trial in a clinical environment. Then, the patients are asked to comment on the fit of this socket. Prosthetists attempt to diagnose potential issues based on patients’ subjective reports, such as rubbing, pain or other discomfort, and address diagnosed problems by modifying the socket. After that, the patients are asked to evaluate the modified socket again. This trial-and-error procedure is often repeated until the patients feel comfortable with the socket. However, the success of this process is heavily reliant on lower limb amputees having the ability to (1) accurately evaluate the quality of the socket fit and (2) communicate any related problems with the prosthetists effectively so the prosthetist can identify potential issues.

Although high expectations are given to 3D printing technologies, the low strength of 3D printing materials and the high printing cost limit its clinical adaptation. One major advantage of 3D printing technologies is that it ensures duplicability of socket design, however, any further modification of the sockets still relies on amputees’ feedback [24, 25]. Comprehensive literature reviews of the design, manufacturing, and fitting of prosthetic sockets can be found in [13, 23, 26].

### Amputees’ Involvement and Challenges

Lower limb amputees are highly involved to avoid potential pitfalls results from poor RLSI function. Amputees are taught to balance fluid intake and body weight, limit excessive perspiration, and frequently inspect the skin for signs of irritation or breakdown [27]. In essence, amputees must be vigilant with respect to any changes in the RLSI [28]. New amputees may be less familiar and less skilled with this monitoring, and vigilance alone does not always result in appropriate responses by prosthetic users, who must first understand the complex dynamics of the RLSI, buffered by extensive real-life experience [29]. Complicating factors, such as neuropathy or vision impairment, can make monitoring even more challenging. Even for an experienced amputee, maintaining vigilance itself can be a heavy mental burden.

## Clinical Needs for RLSI Monitoring

### Standard Clinical Procedure for a New Socket (in the US)

Because the prosthetic socket is the key component of an RLSI, the life span of RLSI is aligned with the prosthetic socket can be categorized into two stages: manufacturing and maintenance. The first stage starts with the patient receiving a prescription for a prosthetic socket and visiting the prosthetist for measurements, and the second stage starts when the patient receives the socket and starts using it during their daily life.

### Manufacturing Stage (Several Weeks to Several months)

**Fig. 2:**
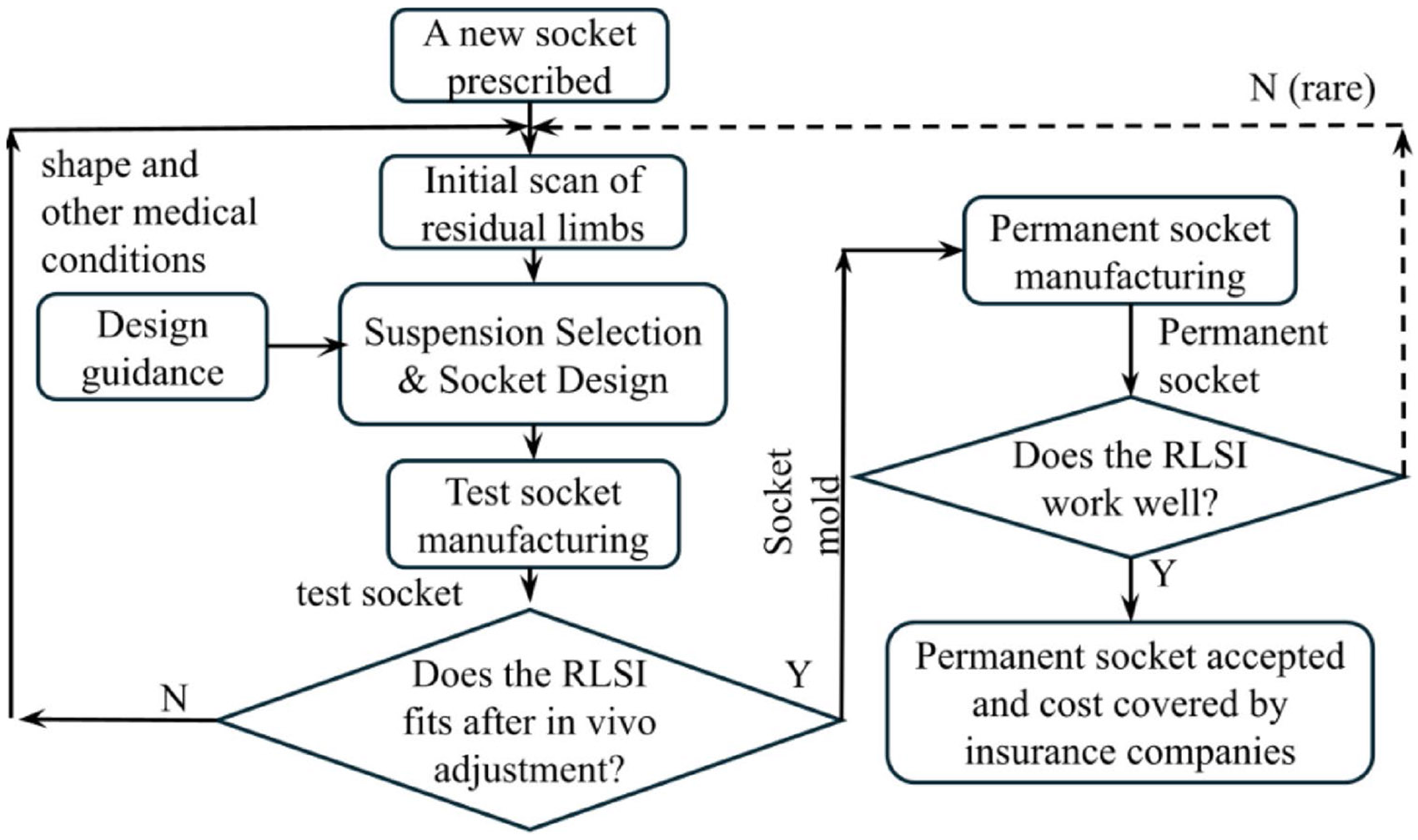
A flow chart for common socket manufacturing procedures

Figure 2 shows the general procedure of manufacturing a new socket. The process starts in the top left corner of the diagram: After a new prosthetic socket is prescribed by a clinician, a prosthetist begins to work with the amputee to produce a new socket. The prosthetist needs to collect detailed geometric information about the residual limb by scanning, measuring, or casting. This geometric information is the foundation of the RLSI construction, including socket design, selection of suspension systems, and selection of related components. Other medical considerations, such as the location of scars or bony protuberances, are also considered. After deciding the suspension system, the socket is often built under the guidance of an established socket design, such as patellar tendon bearing and patellar-tendon-supracondylar [13, 30]. Recent developments, which focus on finite element analysis based socket design [31, 32] and design customized based on patients’ residual properties [33], have not been widely adopted in clinics yet.

Often called a “check socket” [34, 35], a test socket is manufactured to test the socket design outcome. The materials used for the test socket, such as polycarbonate and polyethylene terephthalate glycol, are (1) transparent, allowing prosthetists to observe the residual limb and visually assess socket fit, and (2) easily deformed when heated, allowing prosthetists to conduct quick onsite socket modifications during the evaluation procedure by applying local heat.

The performance of the RLSI based on the test socket is evaluated on patients to verify that (1) the socket is easy to don/doff, (2) the suspension system provides a reliable mechanical connection between the residual limb and the prosthesis, and (3) the patient feels comfortable with the socket. If the RLSI fails the evaluation even with significant modifications, further design, testing, and evaluation must be performed until a well-fitting test socket, which enables a comfortable RLSI, is achieved. Limitations in financial reimbursement to prosthetists often put prosthetists under pressure to reach a good RLSI using a minimum number of test sockets, normally two or fewer.

Once a successful test socket is created, the prosthetist uses it as a mold to make a permanent socket with greater durability. Although the RLSI may undergo changes at this stage, significant revisions of the sockets are more difficult and may not be reimbursed by private health insurance, except when there are documented changes in the shape of the residual limb. After the final verification of the RLSI, the amputee accepts the permanent socket, and the prosthetist files the paperwork and final invoice.

### Maintenance Stage

Once the amputee accepts the permanent socket, the maintenance stage begins. In this stage, components of the suspension systems can be changed, socket redesign halts, and the amputee must rely on the current socket and suspension system to tolerate short- and long-term residual limb changes. Although there is no major modification of the socket, small incremental changes are routinely applied on the socket and suspension system.

In the maintenance stage, almost all the interventions are initiated by the amputee. Amputees conduct routine monitoring of their residual limbs and use prosthetic socks, linerliners, and pads for small adjustments of the RLSI. Even taking the socket off temporarily can have some effect on the residual volume [36]. If amputees feel that these small adjustments are unable to address socket issues or if they experience skin issues, they must reach out to prosthetists and clinicians for help. Prosthetists and clinicians can then conduct more aggressive modifications of existing sockets. These adjustments include changing the local shape of the socket by mounting permanent pads and drilling holes on the socket to reduce the local rigidity of the socket [37]. When the prosthetists and clinicians conclude that no further modification can reestablish the RLSI based on the current socket to fit the needs of the amputees, the maintenance stage ends, and a new manufacturing procedure starts.

Prosthetists and amputees work together to prolong the maintenance stage as much as possible. For amputees, a longer maintenance stage means delaying the return to the long and costly manufacturing stage, maintaining the functionality of the RLSI, and reducing the impact of skin issues. For prosthetists, a long maintenance stage boosts customer confidence and enhances long-lasting personal bonds.

### Existing Approaches to Improve RLSI and Their Limitations

The importance of a reliable and comfortable RLSI is well understood, and efforts to improve the RLSI are conducted at every step of the manufacturing stage. Various approaches have been used to collect geometric information of the residual limb with higher accuracy and reliability [38]. Advanced manufacturing procedures are adopted to increase the accuracy and flexibility of sockets, which are critical for the success of a prosthetist [39-41]. The introduction of a gel liner and various suspension mechanisms results in better tolerance of residual volume changes and more reliable connections between the residual limb and prosthetic sockets [12, 42]. However, none of these approaches alone can ensure a good RLSI.

As mentioned above, test sockets are the key validated tools for verifying socket fitness in vivo; however, this procedure has its own limitations. Although the test sockets allow prosthetists to visualize the interface between the socket and residual limb, the limitations include the following: (1) the test socket fit is unable to provide information during locomotion, where the residual limbs experience the highest load; and (2) the fitting result reflects only the fitting condition when the patient is in the clinic. Currently, some prosthetists allow amputees to bring test sockets home for prolonged tests, this practice does not guarantee clinical advantages and can expose amputees to additional fall risk due to the limited durability of test sockets [43].

Further efforts are also being made to improve the RLSI in the maintenance stage. An inner socket is often included in the socket design and attached to the internal surface of the socket [44]. Made of strong and lightweight materials (softer than the rigid socket), the inner socket increases the flexibility for prosthetists to adjust during the maintenance stage. If the volume of the amputee’s residual limb is reduced, pads can be inserted between the socket and the inner socket so that the size of the prosthetic socket can be adjusted without a new socket. The inner socket can also maintain continuous contact between the residual limb and socket when part of the socket is cut away to release local pressure.

Some prosthetists go further and introduce various mechanisms to permit self-adjustment of socket volume by the amputee themselves [37, 45]. Although these designs provide high flexibility for amputees to deal with residual limb volume changes and making don/doff easier tasks, they still rely on amputees to decide what is the best setup based on amputees’ own feelings [46]. Some automatic adjustable sockets have been built, but their clinical impacts are still not clear [47, 48].

Although it is reported that elevated vacuum suspension systems are able to minimize residual volume changes [11, 49], these suspension systems are difficulty to don/doff and can reduce the range of motion at the knee (for transtibial amputees). Currently, the adaptation of this suspension system is still limited.

### General Challenges in Ensuring a Reliable RLSI

In a summary, the issues related to the RLSI can be summarized as:

- *Changes of residual limbs volume*: Despite all the effort invested in the manufacturing stage, the fit of the socket is expected to deteriorate when the volume of the residual limb changes. Because these volume changes can be caused by various factors, such as body weight changes, pregnancy, or even repeated loads [50, 51], the RLSI deterioration is often unpredictable.
- *Limited resources*: Out-of-pocket payments or private health insurance policies control how much clinical time and resources can be put into socket manufacturing. Prosthetists are forced to balance financial feasibility and amputees’ long-term health. The fixed gap between two manufacturing procedures (usually 6 months) limits prosthetists’ ability to handle problems that cannot be addressed without socket redesign.
- *Lack of quantitative criteria for the RLSI evaluation*: Existing clinical technologies cannot generate accurate measurements of RLSI performance. As a result, prosthetists rely on their own experience and intuition instead of quantified clinical evidence. Socket design and modification therefore become a craft as much as a science [52].

## Key Clinical Decisions

**Fig. 3:**
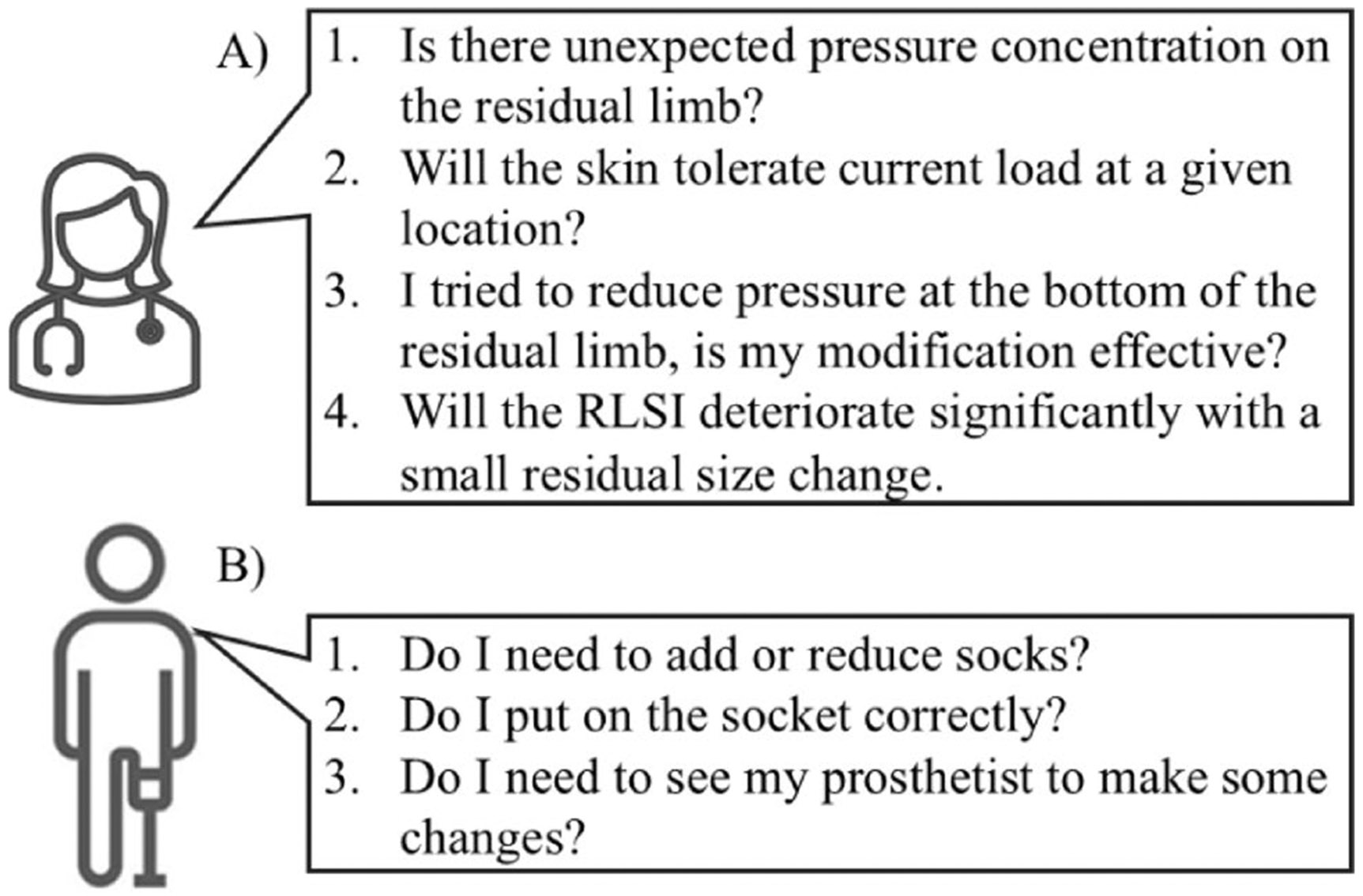
Common clinical questions asked by clinicians (**A**) and amputees (**B**)

Here we would like to highlight unaddressed key clinical challenges of clinicians and amputees in the current procedures related to RLSI. Clinicians want to ensure that (1) mechanical loads during locomotion are distributed appropriately without unexpected pressure concentrations, (2) the load will not lead to skin damage around pressure-sensitive areas or where previous skin issues are reported, and (3) socket-suspension systems can tolerate everyday short-term residual volume changes. Amputees want to ensure that (1) self-management is conducted effectively when it is needed and that (2) clinicians are engaged when the RLSI problem is beyond the scope of self-management. Some of the more specific questions can be seen on Fig. 3.

## Opportunities of RLSI Monitoring

One of the key challenges in generating and managing the RLSI is that most potential interventions rely on amputees to sense changes in the RLSI and report these changes effectively to clinicians [53]. Even for patients with preserved sensation, it may take years and multiple sockets to iteratively develop relevant experience and identify socket fitting issues appropriately. Patients with neuropathy face further difficulties due to their low touch sensitivity. New solutions for amputees and clinicians are needed to quantitatively evaluate the RLSI to improve the comfort and reliability of the RLSI and improve amputees’ quality of life [27].

Engineers and researchers have seized this opportunity to develop various sensing systems for the RLSI and evaluate them with the assistance of amputee participants. These sensor systems target mechanical loads [54-56], physical motion [24, 57], microvasculature [58], bioimpedance [59], and other environmental factors, such as temperature [60, 61] and humidity [62, 63]. Some groups go even further to make automatic self-adjustable sockets [37, 64]. A more comprehensive review of these sensing systems can be found in [65-68].

## Challenges for Quantitative RLSI Monitoring

Although both clinicians and amputees could benefit from reliable, quantitative information about the RLSI, clinical application of RLSI monitoring systems is rare. To demonstrate clinical potentials of RLSI monitoring systems, significant challenges still need to be overcome. Here, we use commercially available pressure measurement pads as an example to demonstrate these challenges [69, 70].

Designed for clinical use, these sensing pads generate sensing grids, which cover a part of the residual limb and can provide accurate pressure readings. However, information from these systems has limited utility in addressing the clinical questions shown in Fig. 3. First, socket design principles encourage prosthetists to rely more on areas that are expected to have high pressure tolerance for weight-bearing tasks; therefore, it is expected that the measured pressure distribution is uneven. By reading the pressure values over a small area on the residual limb, it is difficult to separate a pressure concentration point from an uneven pressure distribution, which is a consequence of following socket design principles. Second, because multiple factors, such as temperature and humidity [71], affect the development of pressure ulcers [72] in addition to the amplitude of vertical pressure, the use of pressure measurements alone to determine the risk of pressure ulcers is not reliable. At the same time, the maximum pressure experienced on the RLSI varies significantly in the literature [73-75], so it is hard to justify whether a measured pressure value is safe or not. In the end, because the pressure pads cover only a small part of the residual limb, prosthetists still need to rely on users’ feedback to verify whether there is a new load concentration caused by the socket modification.

Consequently, these sensing pads can address only question 3 (in Fig. 3A) directly and must be combined with other information sources to address other questions. The benefit of the qualitative measurement is overweighed by (1) the burden of cable management, (2) the efforts to don/doff, and (3) difficulty in registering the pressure distribution back to the residual limb.

### Key Barriers for Clinical Applications

Based on the authors’ experience and understanding, the following barriers are critical. Without addressing them, developed monitoring systems will face challenges when they are translated into clinical applications and win acceptance from clinicians.

### Lack of Guidelines to Support Decision Making in Existing Clinical Procedures

Clearly, objective information alone does not enable evidence-based clinical decision making. Clinicians need detailed guidelines to integrate quantitative measurements with their decision-making procedures. Without these guidelines, clinicians will face difficulty interpreting the collect data and related data collection procedures become waste. Currently, the guidelines related to RLSI are often problematic. Although there is a ¼ inch threshold for pistoning, it is only defined for new sockets and the clinical measured pistoning levels are often significantly larger than this threshold [76]. Suggested pressure distribution on the residual limbs is never quantitative, and often impractical to be personalized. Considering the high variance among amputee population, it is also not very feasible for clinicians to develop their own practical guidelines.

### Limited Resources

As of 2024, the cost of an RLSI based on a new prosthetic socket is approximately $3,000–$12,000 in US dollars and varies based on location, what suspension system is used, and how the socket is designed and manufactured [77]. Although most health insurance policies cover prosthetic sockets and related suspension systems, patients still frequently need to pay out of pocket for deductibles, copays and uncovered components. As prosthetic sockets are replaced after 3 years of usage, on average [78], adding expensive hardware is a financial strain for amputees as well as health insurance payors. This is especially true for systems designed for everyday monitoring. Any high-cost systems that cannot extend the useful life of socket (the most expensive component of an RLSI) are less attractive options for payors unless that cost is offset elsewhere. Amputees already face financial burdens and usually do not have the resources to cover any additional expenses.

Monitoring systems designed for clinical usage are less cost sensitive; however, their success also requires that (1) setting and usage of the system are easy, (2) the measurement results can be interpreted swiftly, (3) most of the system is reusable, and (4) it is compatible with a variety of RLSIs with different socket designs and suspension systems. Among these requirements, the first two protect the precious clinical time of each patient, and the last two ensure that this monitoring system can be operated at a relatively low cost [53].

## Practical Challenges

RLSI monitoring also faces some practical challenges, which are not often considered by researchers at the early stage of development. Although these issues are not obvious in concept validation stage, they affect the reliability and long-term usability of the monitoring system.

### Unexpected Consequences of Monitoring

Monitoring the RLSI itself can potentially lead to unexpected consequences, including additional risk of pressure ulcers or changes in the load distributions. The well-accepted “full contact” principle for prosthetic socket design [13] states that there should be no space between the residual limb and the prosthetic socket. In this way, the full surface of the residual limb can contribute to the weight-bearing effort. Applying this principle leads to the reality that the space between the residual limb and the prosthetic socket is minimized by design. Although widely applied gel liners add a soft layer between the residual limb and the socket, the tight space that results means that any inserts can affect the distribution of mechanical loads. Similarly, drilling holes on the rigid socket surface can also generate unexpected pressure redistributions.

If an RLSI monitoring system relies on inserted sensing elements and holes to access the RLSI, the influence of this monitoring system on the pressure distribution of the residual limb cannot be fully ignored. For long-term monitoring, it is critical to evaluate whether the sensing elements increase the risk of future pressure ulcers or affect the function of the prosthetic sockets. For short-term monitoring in clinics, understanding the impact of the sensing elements on the load distribution will also be helpful for evaluating the reliability of the monitoring results.

### Connection Challenge

The highly dynamic loading conditions inside the sockets make it difficult for any electronic circuit to survive for a long period of time; thus, most RLSI monitoring systems keep only the resilient sensing elements inside the socket and leave the other more fragile components outside the sockets. Then, generating an ergonomic connection between the sensing elements and other electronic components becomes a problem. Although advanced electronic design permits wireless communication [79], traditional cable connections are dominant in the reported literature [55, 56, 63].

Arranging the cable to penetrate the socket shell directly is not recommended because (1) a hole in the socket can damage the integrity of the socket [80] and compromise vacuum formation between the residual limb and the prosthetic socket, and maintaining this vacuum is critical for the success of some suspension systems [81]; (2) it practically fixes the measurement location, and additional holes may be needed if monitoring needs to be conducted at different places; and (3) it violates the full contact principle and can generate unnecessary load concentrations and increase the risk of pressure ulcers [13].

Most existing systems pass their cables through the top hole of the socket [55, 69]. Although this maintains socket integrity, long cables are exposed inside the socket, increasing the risk for electronics failure and possibly forming air pathways, which makes maintaining inner-socket vacuums challenging [82]. Arranging the cables between the inner socket and the socket is a more reliable solution, but this solution can be used only on permanent sockets (not on testing sockets) and is difficult to adjust after the inner socket is finalized.

### Challenges for Long-Term Monitoring

The information gathered in the clinical environment does not fully reflect what amputees face in everyday life during locomotion and provides ecological validity. However, bringing a monitoring system for routine usage in their daily life environments requires additional efforts from amputee users, who are already often burdened by other health-related issues. For RLSI monitoring systems, the following factors must be considered for amputee compliance: the systems must (1) be comfortable to use; (2) provide reliable information that can help guide intervention [83]; (3) enable personalized care; and (4) ensure collaborative care.

These monitoring systems must be designed and managed not only at the intervention level (how patients use this system) but also at the intrapersonal level (preparation of patients through education), the interpersonal level (communication between clinicians and patients), and the organizational level (coordination among clinicians and administrators to maximize the impact of the system) [84].

## Design of RLSI Monitoring Systems to Meet Clinical Needs

During the design phase, aligning RLSI monitoring systems with existing clinical decision-making processes offers several clear advantages. First, it allows the clinical benefits of the monitoring systems to be more easily demonstrated. Second, it provides a straightforward pathway for integrating these systems into routine clinical workflows. Finally, the same procedures used to validate the monitoring systems can also serve as a foundation for developing formal clinical guidelines. Here, we identify unaddressed clinical needs and recommend potential technical solutions based on our own experience of related challenges.

### Full Residual Limb Load Monitoring to Verify Test Sockets

Analyzing the mechanical loads distributed across the entire residual limb is essential for prosthetists to ensure proper load balance and socket fit. This analysis is particularly important during the evaluation of a test socket. To support this task, effective monitoring systems should meet the following criteria: (1) provide coverage across most of the residual limb surface to help detect unexpected pressure concentrations, (2) integrate seamlessly with existing RLSI systems to minimize installation effort, and (3) register measurement data directly on the residual limb to enable intuitive interpretation by the prosthetist.

While some systems have demonstrated automatic data registration on the residual limb [85], a more straightforward approach leverages the transparency of the test socket—allowing pressure readings to be visualized directly on the limb. To enable this, researchers may consider developing soft materials that: (1) mimic the mechanical properties of gel liners or prosthetic socks so they can replace these components during the test socket fitting process, and (2) exhibit optical changes in response to mechanical loading, allowing load distribution to be observed in real time without the need for additional instrumentation.

**Fig. 4 ,:**
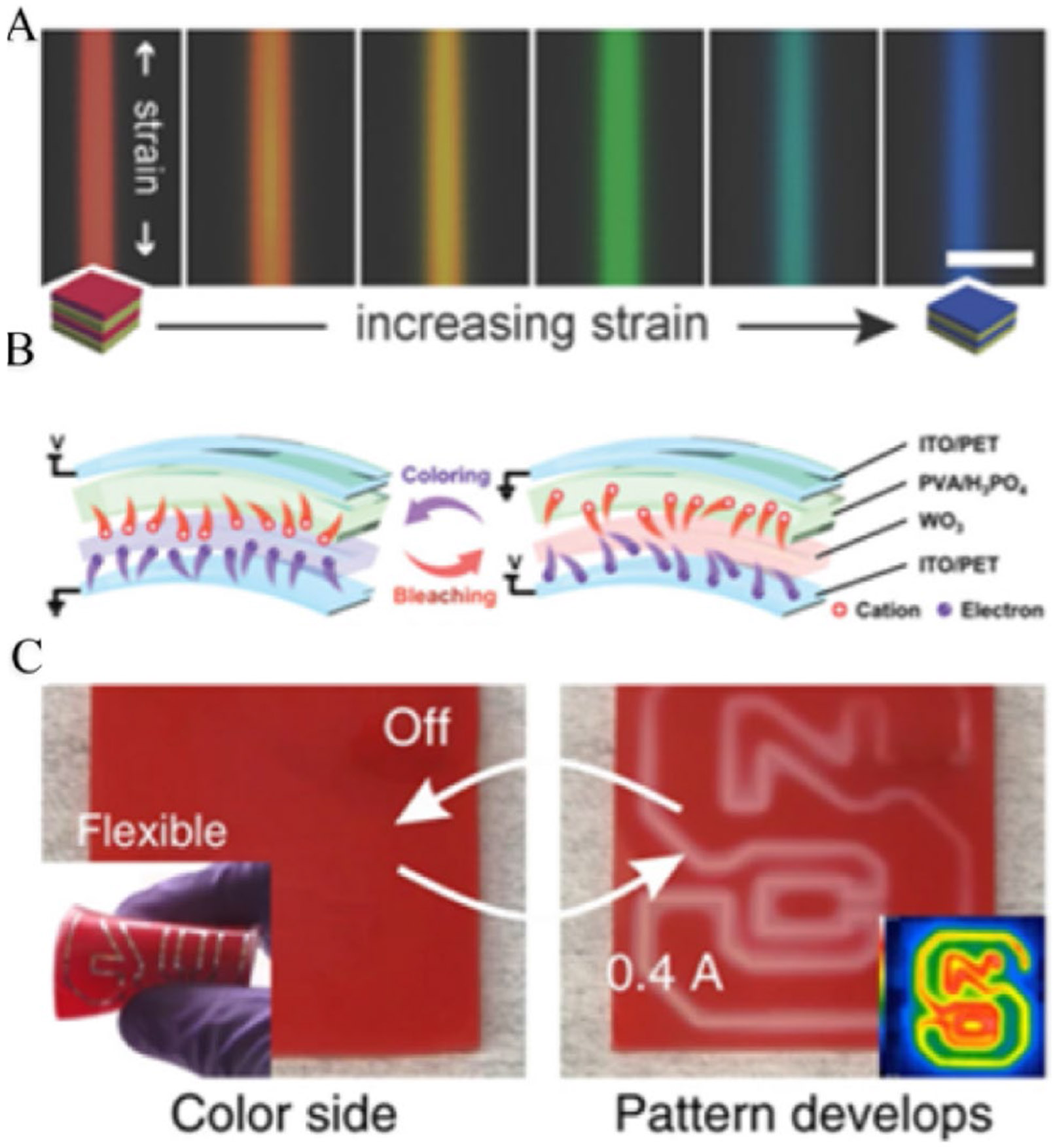
materials, which change colors under mechanical loads. **A** Stretchable optomechanical fibers under different strains. **B** Schematic of the redox reactions in a pressure sensing system. **C** Thermochromic-based sensing system. The image at the right corner was taken with an IR camera to show temperature changes

Sandt et al. demonstrated that stretchable optomechanical fibers could present different colors in response to the various strains applied to the leg without any power source (Fig. 4A) [86]. An elastomeric photonic multilayer structure, where hundreds of nanometer-thick layers of polydimethylsiloxane (PDMS, silicone) and polystyrene-polyisoprene triblock copolymer (PSPI) intersect, could act as a Bragg reflector with different colors according to the strain. When the fiber is stretched, its reflection peak shifts from longer wavelengths to shorter wavelengths, moving from the red end of the spectrum to the blue end. The connections among strain, pressure, and the resulting color change suggest the potential for developing a pressure sensor. Because the strain in the fiber is directly related to the external load, color changes may occur very quickly, which makes it more difficult to read during locomotion when the load change can be dramatic.

More work has been done to generate soft materials whose electronic properties, such as capacity and conductivity, can change with an increasing mechanical load [87-89]. To visualize these electronic property changes, additional physical or chemical procedures are necessary, and additional external energy sources are needed to drive these physical or chemical procedures.

Redox reactions can be used to visualize local capacity changes (Fig. 4B). Guo et al. generated a sandwich structure with pyramid-like microstructures in a polyvinyl alcohol (PVA)/H3PO4 layer and a WO_3_ film between two indium tin oxide/polyethylene terephthalate (ITO/PET) electrodes [90]. When pressure is applied to this sandwich structure, the contact area in the (PAV)/H_3_PO_4_ layer changes, which affects the capacity between the two electrodes, as does the number of electrons and cations (H^+^) on the electrodes. These electrons and ions participate in a redox reaction within the WO_3_ film, which changes the color of the film from white to blue when compressed. By controlling the power supply to the sensing system, it is possible to lock the sensor reading at a specific time. Exposing users to WO_3_, a toxic material, may lead to additional risks.

Thermochromic materials can be used to visualize local conductivity changes. Jin et al. demonstrated a soft and thermochromic PDMS layer with embedded liquid metal microchannels (Fig. 4C) [91]. When consistent current is applied through the microchannel, heat induced by Joule heating is generated along the microchannel. When the sensor is pressed, the microchannel in the compressed area becomes narrowed with reduced conductivity (increased resistance). Because the current is constant, the increase in local resistance leads to increased Joule heating and causes the local temperature to rise. The temperature change is then displayed by the color change of the PDMS due to the embedded thermochromic materials, as shown in Fig. 4C. Because the local temperature change is the result of heat accumulation, the observed color change reflects the average pressure at the measurement point only.

### Estimate the Risk of Skin Issue at Given Locations

Another critical task for prosthetists is assessing whether a socket may cause pressure ulcers at vulnerable areas, such as scar tissue or sites with a history of skin breakdown. Since it is well established that mechanical load alone does not determine the development of pressure ulcers [71], this type of prognostic decision must be guided by biomarkers that more directly reflect skin health.

Pressure ulcers are thought to be caused by ischemia around the area under extensive pressure [92]. Thus, monitoring microcirculatory occlusion or tissue oxygen levels is a rational solution for evaluating the likelihood of pressure ulcer development. Common in vivo approaches include transcutaneous oxygen measurement [93], metabolic positron emission tomography [94], near-infrared spectroscopy [95], and transcutaneous oxygen measurement [96]. However, these approaches are usually evaluated under static loading conditions, and little is known about their performance under periodic loading conditions. At the same time, they also face the challenges mentioned in the previous session.

Technologies that permit clinicians to detect pressure ulcers in their early stages have been explored, and some technologies, such as subepidermal moisture (SEM) scanners, thermography, and ultrasound, are commercially available [97]. However, applying these technologies in the socket manufacturing stage is a practical challenge. If a pressure ulcer is detected, the patient must wait for it to heal before they can test the socket again; otherwise, the prosthetist will be unable to determine if the observed pressure ulcer is caused by the current socket setup or previous one. This delay prolongs the costly socket manufacturing procedure. However, early-stage pressure ulcer detection might be used as a final check of socket fitness.

### Information to Support Amputee-Driven RLSI Self-management

Unlike clinicians, who are trained in socket design and fitting principles, amputees typically lack this technical knowledge and prefer simple, experience-based indicators to evaluate their prosthesis. One such indicator is pistoning—the relative movement between the residual limb and the socket during walking—which is widely used in clinical practice and is generally understandable to amputees.

Because most amputees wear gel liners, practical pistoning tracking methods often measure the relative motion between the liner and the socket. These methods rely on the unverified assumption that the liner remains in constant contact with the residual limb and moves with it uniformly at all locations during gait. Although there is limited scientific evidence to support this assumption, researchers often adopt it to avoid the significant challenge of directly placing and tracking markers on the residual limb skin in everyday use.

**Fig. 5:**
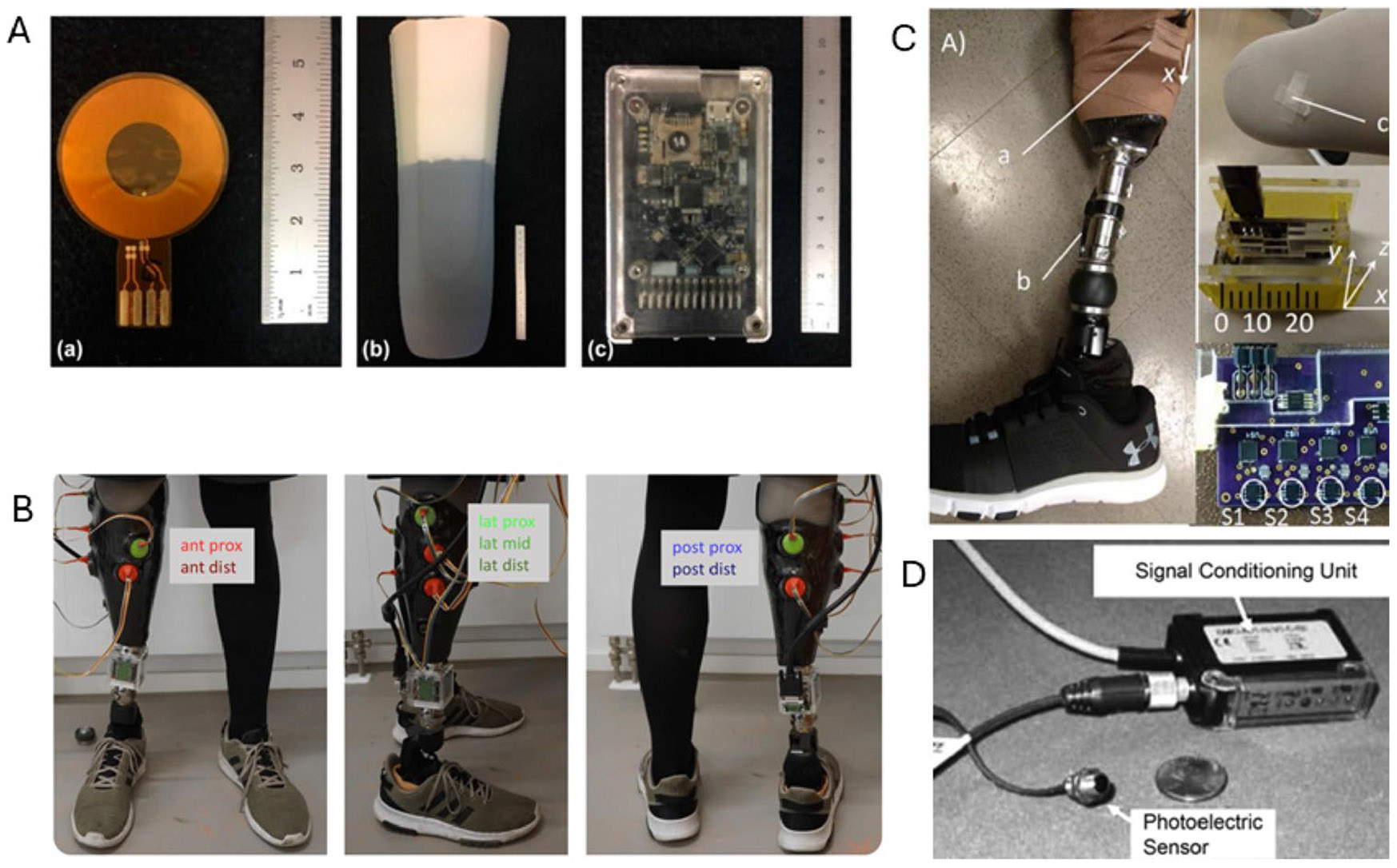
Examples of pistoning monitoring systems. **A** The system, which monitors the motion perpendicular to the residual limb. **B** Optical sensing for pistoning. **C** Magnetic tracking for pistoning. **D** Track pistoning by measuring the distance between the distal end of the residual and the socket (one of the earliest designs)

In most existing pistoning monitoring systems, the pistoning is evaluated by measuring the relative motion between the prosthetic socket and one trackable marker, which is attached to the residual limb. The markers can be a part of the existing prosthetic component tree or a small insert into the socket suspension system. Some examples include pin of the liner [98], bottom of the residual limb [99] (Fig. 5D), socks with special magnetic properties [100, 101] (Fig. 5A), and small magnets [102] (Fig. 5C). These markers are chosen with the following considerations: (1) minimizing the efforts of donning and doffing and (2) avoiding potential pressure concentration caused by the marker.

Another approach is to measure the relative motion between the liner and the socket directly. Highly accurate optical sensors, which are also often used in optical computer mice, are used to track surface patterns of the liner so that the relative motion between the liner and socket can be tracked. This approach does not rely on a specific tracking marker and has been demonstrated in [103] (Fig. 5B). The limitation of this solution is that the optimal sensor needs to be integrated in the socket (usually through a hole on the socket) to ensure that it can access the liner surface directly.

A common problem with pistoning monitoring is to define an acceptable threshold to distinguish normal pistoning from abnormal cases. Although the rule of thumb for prosthetists is only ¼ inch [104], large variations in pistoning have been reported in the literature. In some cases, patients can tolerate more than 1 inch of pistoning without obvious skin damage [57]. The only exception is the pistoning perpendicular to the surface of the residual limb, which is expected to be zero based on total contact theory [105]. However, this pistoning motion is difficult to measure, and the related literature is limited to understanding the properties of this motion.

Furthermore, pistoning can vary significantly across different locations on the residual limb [57]. Existing single point pistoning monitoring programs should be regarded only as a very narrow view of the big picture. Although information from these systems may reflect the general trends of changes in socket fitness, such information helps little in guiding permanent socket changes or socket redesigning.

### Establish Personalized Clinical Guidance

While it is difficult to develop universal clinical guidelines for RLSI management due to the high variability among amputees, it is still feasible to establish personalized guidelines. For example, defining a single maximum safe pressure threshold that applies to all amputees may be unrealistic. However, for an individual patient, it may be possible to identify a pressure threshold above which the likelihood of pressure ulcers increases. This type of personalized knowledge could be developed by continuously monitoring the residual limb over an extended period, capturing the dayto-day variations in the RLSI and correlating them with skin health outcomes.

To enable a long term monitoring system for lower limb amputees, the sensing elements need to (1) cover the majority area of the residual limb and (2) be integrated into an existing prosthetic component, because minimizing exertion and cost needs are critical for the success of any long-term monitoring approach [53]. Compared with liners, which are costly to replace, prosthetic socks are much less expensive and can serve as a platform to integrate long-term RLSI monitoring systems. Textile sensing technologies have been developed to integrate sensing elements and electronic conductors into textile products.

Existing technologies often rely on piezoresistive, capacitive, piezoelectric, and triboelectric properties of textiles to sense changes in pressure [106]. Piezoresistive sensing provides high detection sensitivity, fast response time, and a large detection range [106, 107], whereas capacitive-type sensors offer good linearity and low pressure detection limits but provide relatively lower sensitivity and require more complex circuitry to operate [70]. Piezoelectric and triboelectric sensing, although less frequently employed, do not require external energy supplies for their operation, making them desirable options for low-power or self-powered sensing applications [106, 108]. However, triboelectric sensing does not work well in moist environments, which limits its utility within the RLSI. Textile sensing systems for tracking wetness, temperature changes, and biopotential signals have also been reported in the literature [109].

**Fig. 6:**
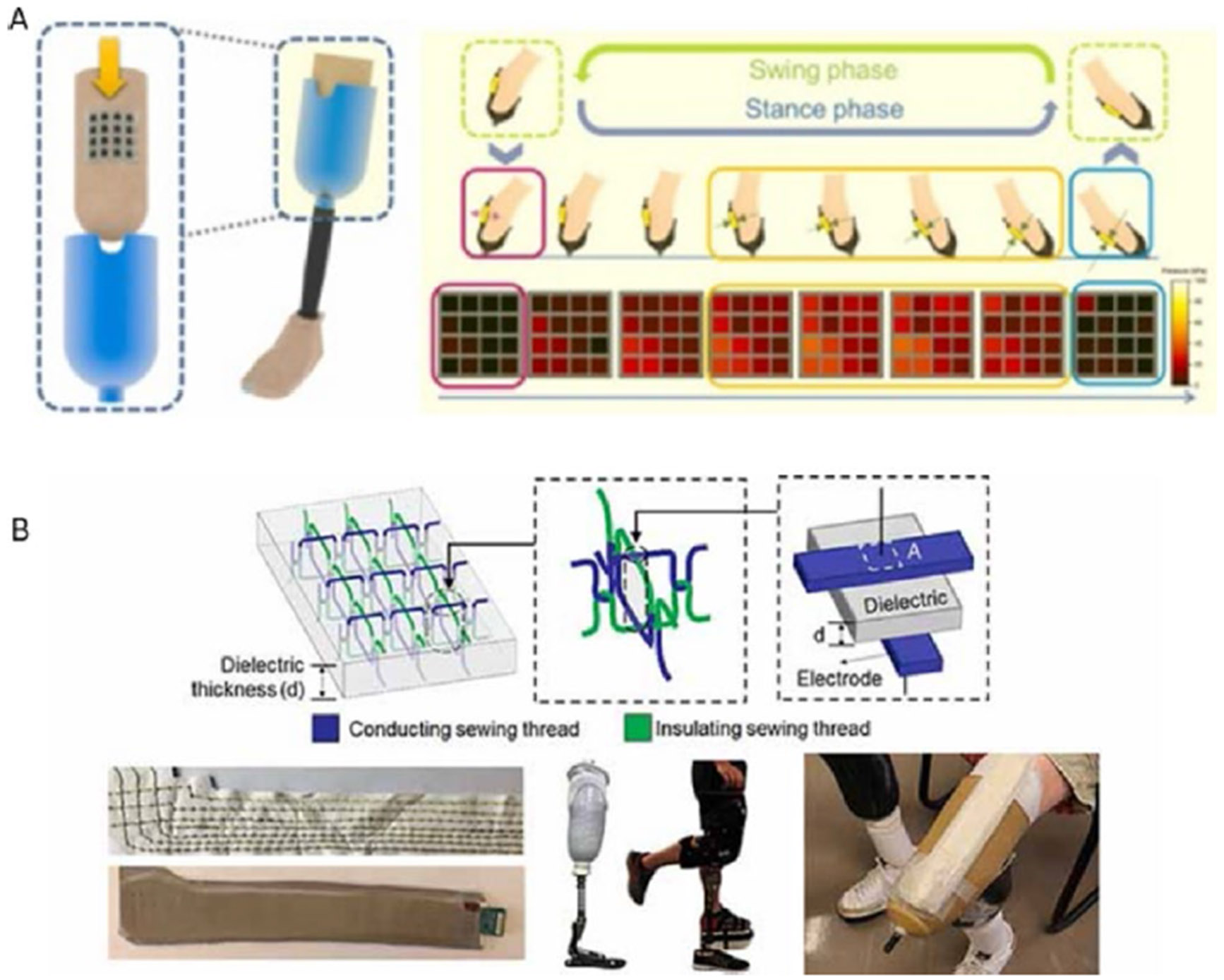
**A** A self-powered triboelectric nanogenerator. **B** Textile capacity-based pressure sensing for ampu-tee sockets

Several textile sensing systems have been developed for residual limb monitoring for lower limb amputees. K.-B. Chang et al. presented a self-powered triboelectric nanogenerator sensor array composed of PDMS and polycaprolactone nanofibers capable of monitoring real-time interfacial pressure distributions. When evaluated with simulated walking within a prosthetic socket, the system demonstrated high stability, high flexibility, high durability, low cost, and insensitivity to temperature, humidity, and curvature (Fig. 6A) [108]. Although not a true textile-based sensor system, the promising results of this approach may serve to guide similar efforts with fabric-based, fiber-based or nonwoven triboelectric nanogenerators, which may be more easily integrated into existing socket interface layers. This system has not yet been tested on amputees.

J. Tabor et al. demonstrated a fully textile-based woven sensor with dedicated data acquisition and communication capabilities for socket fitness monitoring (Fig. 6B). This work presents a system consisting of four discrete, capacitive-type sensor array patches, each formed through perpendicularly sewn seam lines of conductive and insulating yarns on a melt-blown fabric dielectric. A 3 × 3 sensing grid of overlapping pressure-sensing “texels” was created and encapsulated within a conductive copper–nickel-coated polyester fabric to provide shielding from environmental electromagnetic interference. Testing across a plaster artificial limb, an able-bodied subject, and an amputee subject demonstrated this approach’s ability to accurately monitor pressure distribution at the anatomical sites of interest (patella tendon and popliteal depression) during body weight shifting and walking [110].

Currently, these sensing systems are mostly confined to laboratory environments. Clinical trials in which amputees use these sensing systems at home are needed to verify the effectiveness and reliability of these systems.

## Conclusion

The residual limb–socket interface (RLSI) plays a critical role in the rehabilitation of lower limb amputees, yet there is a lack of objective methods to evaluate its performance. Although various sensing technologies have been developed to capture detailed information about the RLSI, their clinical impact remains limited. This perspective aims to: (1) summarize current clinical procedures related to the RLSI, (2) highlight unresolved clinical questions, (3) outline key challenges in RLSI monitoring, and (4) identify technologies that can be more readily integrated into clinical decision-making workflows. Through this discussion, we aim to encourage sensor designers to (a) align their efforts with clinical needs, so their innovative work can lead to direct clinical impacts, (b) consider all the known practical challenges to reduce the chance of design failure, and (c) demonstrate their success based on clinical outcomes instead of bench test only. We hope this work will trigger more translational research efforts which can bring RLSI monitoring systems into routine clinical practice.

## Data Availability

No datasets were generated or analysed during the current study.
